# The influence of structure and local structural defects on the magnetic properties of cobalt nanofilms

**DOI:** 10.3762/bjnano.14.3

**Published:** 2023-01-04

**Authors:** Alexander Vakhrushev, Aleksey Fedotov, Olesya Severyukhina, Anatolie Sidorenko

**Affiliations:** 1 Modeling and Synthesis of Technological Structures Department, Institute of Mechanics, Udmurt Federal Research Centre, Ural Division, Russian Academy of Sciences, Baramzinoy 34, Izhevsk 426067, Russia; 2 Orel State University named after I.S. Turgenev, Komsomolskaya Str. 95, 302026, Orel, Russia; 3 Nanotechnology and Microsystems Department, Kalashnikov Izhevsk State Technical University, Studencheskaya 7, Izhevsk 426069, Russia; 4 Institute of Electronic Engineering and Nanotechnologies of Technical University of Moldova, Academiei 3/3, Chisinau 2028, Moldova

**Keywords:** LAMMPS, magnetic materials, molecular dynamics, nanocomposites, nanofilms, spintronics

## Abstract

The present paper considers a mathematical model describing the time evolution of spin states and magnetic properties of a nanomaterial. We present the results of two variants of nanosystem simulations. In the first variant, cobalt with a structure close to the hexagonal close-packed crystal lattice was considered. In the second case, a cobalt nanofilm formed in the previously obtained numerical experiment of multilayer niobium–cobalt nanocomposite deposition was investigated. The sizes of the systems were the same in both cases. For both simulations, after pre-correction in the initial time stages, the value of spin temperature stabilized and tended to the average value. Also, the change in spin temperature occurred near the average value. The system with a real structure had a variable spin temperature compared to that of a system with an ideal structure. In all cases of calculations for cobalt, the ferromagnetic behavior was preserved. Defects in the structure and local arrangement of the atoms cause a deterioration in the magnetic macroscopic parameters, such as a decrease in the magnetization modulus.

## Introduction

The analysis of phase transitions and related critical phenomena in condensed media is a complex, time-consuming, and often a high-cost process from a technological point of view [1–3]. On the one hand, this is due to the need to use a comprehensive approach in theoretical studies, since the behavior of different phases is often described by different models or state equations [4]. Another reason is that phase transformation mechanisms originate at the nanoscale and atomic levels [5–6], where observation and experiments require modern and expensive equipments. In this regard, precision experimental studies in critical regions are fraught with significant difficulties due to both temporal and spatial scales of object behavior [4].

Despite the existing difficulties, the interest in the study of phase transitions is not decreasing. Evolutionary analysis of the structural transformations of substances finds wide application in many areas of science and technology, including physics of multicomponent systems. One promising application of multicomponent systems is the development of phase-transition heat-storage materials [7–8], in which heat storage and accumulation occur due to phase transformations. The functioning of such storage media is based on energy fluctuations in the process of crystallization or melting of the media. In contrast to traditional media, thermal storage does not require sealing of the working volume during change of aggregate states, and is actively implemented as a highly efficient and energy-saving technology in the field of construction [9] and solar energy [10].

Phase transformations occupy an important position in the theories of superconductivity and ferromagnetic alloys. These theories actively consider composites with shape memory [11–12]. Such composites are also called intelligent materials of the future [13] due to their unique functional properties and the possibility of restoring the original parameters under certain external conditions. Both thermodynamic conditions [14] and magnetoelectric fields [15] can act as external perturbations affecting the internal state and phase transitions of the samples. It has been shown in [11–12,16] that structural phase transitions in shape memory materials are in close relationship with external static and induction fields. Studying the role of magnetism on the structural features of composites opens up promising possibilities, since it allows predicting and creating new materials with controllable properties.

The idea of mutual correlation between material structure and its magnetic properties is being developed in the field of spintronics. Modern computing devices face a number of difficulties during production, including those related to arrangement of nanoscale computing elements on integrated circuits and their subsequent cooling during operation [17–18]. Problems related to excessive heat dissipation and performance improvement can be solved with the help of spintronics devices, which are currently presented in a fairly wide variety of valuable effects: spin valves and valves in thin films and heterostructures [19–20], sensors based on the anomalous Hall effect [21], spin injection and magnetism detection [22–23], giant magnetic resistance effects in data storage items and hard drives [24–25], ultrafast magneto-optical switches and optically induced ferromagnetic materials [26]. The discovery and implementation of topological insulators in Josephson contacts make spintronics devices excellent candidates for applications in quantum computing [27–28] as well as in quantum cryptography [29].

The extensive influence of phase transitions and critical phenomena on the working properties of the samples testifies the importance of a detailed study of structural transformations and possible stable states. Morphological analysis enables the identification of local defects in the crystal structure, which form different scale aggregates that can further serve as causes of deterioration of the target material functional characteristics [30–31]. Comprehensive studies in this area not only allow to establish the presence of structural heterogeneities and features, but also to formulate the main laws of their origin and development.

This work is devoted to solving an important problem regarding the relationship between the magnetic properties of multilayer nanocomposites and their structure. The problem of studying the influence of structure on the materials magnetic properties is not new and has been previously solved by other authors [4,32–34]. For example, in [4], to describe the thermodynamic equilibrium and nonequilibrium properties of magnetic materials, a multiscale approach of a mathematical model is used. This approach includes methods of first principles, spin models based on the stochastic Landau–Lifshitz–Gilbert equation, and a submodel of micromagnetism, described by the Landau–Lifshitz–Bloch equation. The reference [32] is also devoted to the development of modeling methods in the field of materials phase transitions, but with the help of classical and quantum Monte Carlo approaches. The main emphasis of the work is placed on studies of the statistical lattice model, including a high-precision calculation of the critical indices.

The intermetallic magnetic compound FeRh is discussed in [33]. In the considered material, the thermodynamic first-order phase transition is observed near room temperature. Heating the material above the transition temperature changes its magnetic behavior from antiferromagnetic to ferromagnetic and is accompanied by a significant change in the crystal lattice structure and an increase in electrical conductivity. The material is promising for applied research and development of new spintronics devices, energy management sensors, and magnetic recording media.

Research focused on specific application devices based on phase-transition memory state is discussed in detail in [34]. Phase-transition memory technology is among actively developing and promising technologies since it enables the design of small devices with high performance, durability, and cost-effectiveness. The authors of [34] review how the characteristics of phase transition memory combine with various potential applications, addressing some of the problems of this technology, including those related to cell design, negative structural features, and changes in nanomaterials that can occur during fabrication.

Thus, the evaluation and elaboration of structural changes in a nanomaterial arising from its production are important tasks, often closely related to the composition of the sample in question. In this paper, we propose one mathematical model to investigate the relationship between the material structure and its magnetic properties. Mathematical modeling is used to estimate the influence of the disturbances in the atomic arrangement inside the crystal lattice, in the destruction and fragmentation zones of spin orientation inside the material, and overall magnetization of the sample.

## Description and Conditions of the Numerical Experiment

The structure and magnetic properties of the nanomaterial were investigated in this work using a promising nanocomposite formed by alternating layers of cobalt and niobium. The proposed composite has potentially promising functional properties and can be used in magnetic systems with controlled effective energy exchange in Josephson contacts [35], which are successfully implemented in memory and information storage devices. A similar layered heterostructure, but with the addition of a thin platinum film necessary for the generation of spin–orbit bonds, is also described in [36].

**Figure 1:**
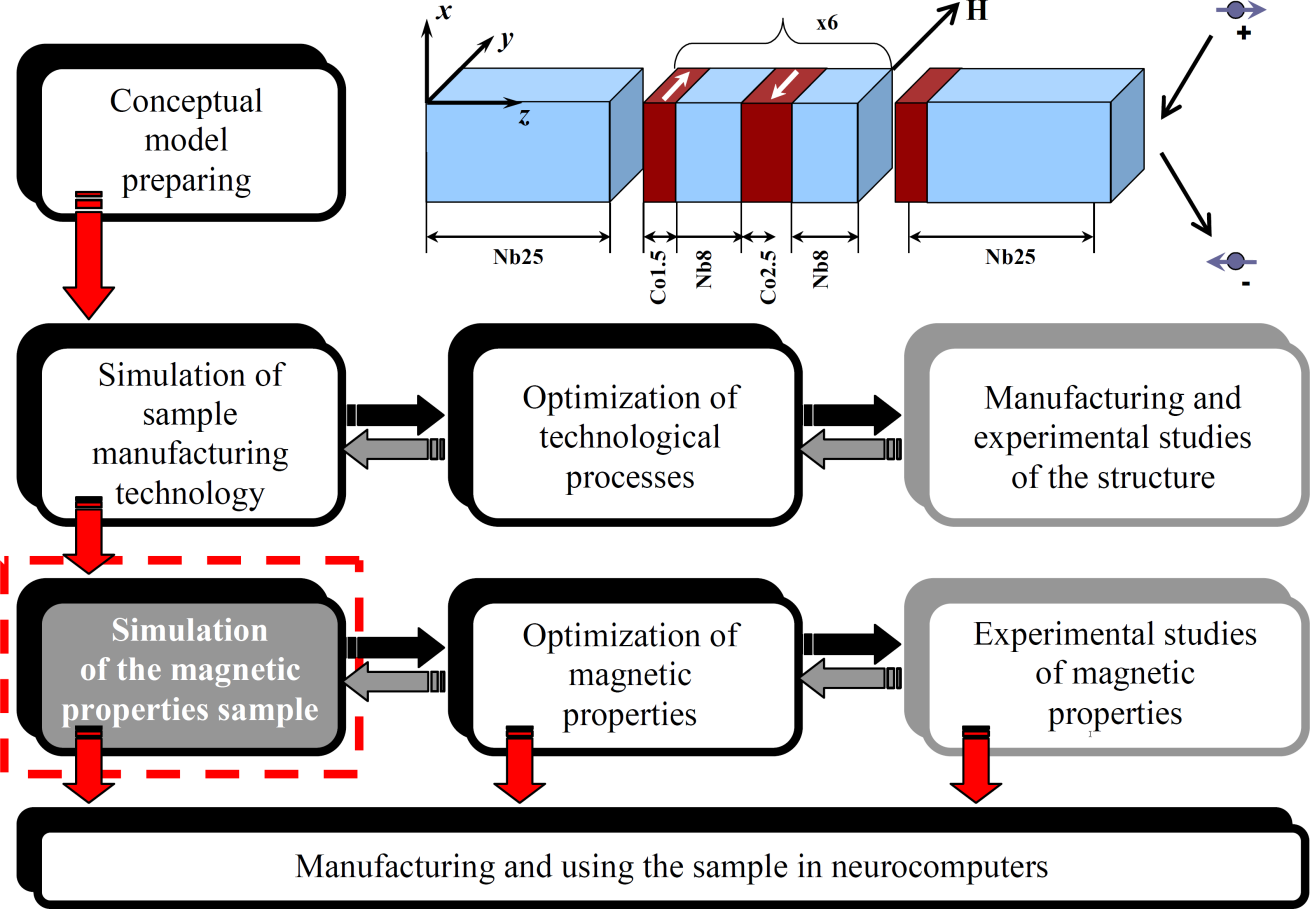
Problem statement for the complex study of cobalt and niobium heterostructures. The sketch of the Nb/Co spin-valve nanosystem was reproduced from [37] (© 2020 A. Vakhrushev et al., distributed under the terms of the Creative Commons Attribution License (http://creativecommons.org/licenses/by/4.0)).

Comprehensive research on new promising materials is a complex multistage process. The general scheme of the problem-oriented analysis of a multilayer composite of niobium and cobalt is presented in Figure 1. At the preparation stage of the conceptual model, the expected requirements to the main properties of the predicted material are formulated, a manufacturing method, and an approximate composition are proposed on the basis of already existing technologies. The conceptual model for our study is based on a sample whose structure and composition is shown in the upper right part of Figure 1.

At the next development stage, the technological processes of nanocomposite manufacturing were simulated and systematized. This was done to establish the dependence of the structure and characteristics of the nanocomposites on the production parameters, to check the presence of target functional aspects, and to determine controllable properties (i.e., those properties that are influenced and corrected in the manufacturing process). The previously conducted studies considered the influence of sample parameters (e.g., temperature of the substrate on which the magnetron sputtering of nanofilms takes place, the intensity and deposition direction) on the final properties of the sample. The results of numerical experiments are described in the literature [37–39].

The next stage of sample study involved the optimization of the nanofilm interface. The basic magnetic properties of the nanocomposite depend on the quality of the interface between the layers, so the problem of obtaining clearly separated contact layers is highly relevant. Using simulations, it was demonstrated that optimization of the nanofilm interface can be obtained either by introducing additional intermediate thin layers neutral to the original composition, such as aluminum oxide, or by additional processing means, such as mechanical alignment and intensive substrate cooling. The stage of experimental studies of the sample structure is necessary to identify the real structure of the nanocomposite and to compare the data with previously obtained simulation results.

This current work is aimed at modeling the magnetic properties of the nanomaterial heterostructure under study. In Figure 1, the block of modeling magnetic properties is highlighted by a red dashed line. As noted earlier, the formed nanofilms have a nonideal structure. Consequently, the influence of the real structure and local order of atoms on parameters with considerable practical interest (e.g., magnetization, different types of energies, spin temperatures, and particle orientations) remains open.

The last two steps of the analysis, which include the optimization of magnetic properties and experimental study of magnetic properties, are the subject of future research and are cited in this work for a complete understanding of the complex task of developing new promising nanomaterials.

## A Mathematical Model for Studying the Magnetic Properties of Nanomaterials

When describing the magnetic properties of a nanosystem, simultaneous equations of classical molecular dynamics are used, which are supplemented by considering the spin vectors **s***_i_* for each atom. The motion equation for atoms and spins is written in the following form:


[1] $$\frac{\text{d}r_{i}}{\text{d}t}=\frac{p_{i}}{m_{i}},$$

[2] $$\frac{\text{d}p_{i}}{\text{d}t}=\sum_{i\neq j}^{N}\left[-\frac{\text{d}U\left(\left|r_{ij}\right|\right)}{\text{d}\left|r_{ij}\right|}+\frac{\text{d}J\left(\left|r_{ij}\right|\right)}{\text{d}\left|r_{ij}\right|}s_{i}\cdot s_{j}\right]e_{ij},$$

[3] $$\frac{\text{d}s_{i}}{\text{d}t}=f_{i}\times s_{i},$$

where **r***_i_* is the vector characterizing the position of the particle *i*; **s***_i_*,and **s***_j_* are the spin vectors; **p***_i_* is the momentum; **e***_ij_* is the unit vector along **r***_ij_*; **f***_i_* is the analogue of the force applied to spin; and *U* is the potential energy.

The general form of the expression for describing the total energy of magnetic systems can be written in the following form:


[4] $$H=H_{\text{z}}+H_{\text{ex}}+H_{\text{an}}+H_{\text{Neel}}+H_{\text{dm}}+H_{\text{me}}+H_{\text{di}},$$

where the first two terms in the right-hand side are the Zeeman and exchange interactions, respectively, the next two terms describe magnetic anisotropy, followed by the terms responsible for the Dzialoshinsky–Moriya, magnetoelectric, and dipole interactions, respectively. The consideration of different types of interactions in a model depends on the structure of the systems considered, as well as on problems that are solved in the simulation. The determination of parameters used to describe different types of interactions in modeling magnetic systems requires additional numerical and experimental investigations. For this reason, the emphasis at this stage was placed on the pairwise anisotropy model of Neel.

The exchange interaction provides a natural connection between the spin and lattice degrees of freedom due to the dependence of the function *J* on the interatomic distance. This function determines the intensity of the interaction. As noted in [40], the function *J* is a symmetric radial function. Due to its symmetrical representation, only isotropic phenomena and processes in materials can be described using the *J* function. At the same time, anisotropic effects are of great interest, since they often affect the most prospective and promising magnetic nanomaterials. Magnetic crystallographic anisotropy arises on spin–orbit interaction of atoms. As a consequence, this type of interaction should be separately taken into account when constructing theoretical models and conducting numerical experiments.

The type and parameters of the crystal lattice of magnets largely determine the type and shape of the resulting magnetic anisotropy. In ferromagnets, magnetic anisotropy is characterized by the magnitude and orientation of the magnetization, as well as by the change in the magnetic energy of the material. The main causes of magnetic anisotropy are temperature changes, dipole interactions, mechanical deformations, or other external factors. If external influences are absent, then due to spin–orbit interactions of atoms inside the nanomaterial, magnetic crystallographic anisotropy can occur, which is caused by a change in the internal energy and by the symmetry or asymmetry of the crystal structure of ferromagnets.

The dipole–dipole interaction does not make a significant contribution to the anisotropy energy and its value is insignificant. Only in a number of rare-earth metals the contribution of the dipole–dipole interaction can be significant due to large magnetic moments of the atoms and small values of the crystal lattice parameters.

Approximations for modeling spin–orbit coupling have been proposed in [41–42]. In particular, the functions proposed by Neel [41] for modeling the bulk magnetostriction and surface anisotropy in cobalt were used in [43]. The model proposed by Neel considers magnetocrystalline anisotropy in more complex forms as compared to uniaxial anisotropy. This model is used to describe magnetocrystalline anisotropy between pairs of magnetic spins:


[5] $$\begin{array}{c} H_{\text{Neel}}=-\sum_{i,j=1,i\neq j}^{N}g_{1}\left(r_{ij}\right)\left(\left(e_{ij}\cdot s_{i}\right)\left(e_{ij}\cdot s_{j}\right)-\frac{s_{i}\cdot s_{j}}{3}\right)+\\ +q_{1}\left(r_{ij}\right)\left({\left(e_{ij}\cdot s_{i}\right)}^{2}-\frac{s_{i}\cdot s_{j}}{3}\right)\left({\left(e_{ij}\cdot s_{j}\right)}^{2}-\frac{s_{i}\cdot s_{j}}{3}\right)+\\ +q_{2}\left(r_{ij}\right)\left(\left(e_{ij}\cdot s_{i}\right){\left(e_{ij}\cdot s_{j}\right)}^{3}+\left(e_{ij}\cdot s_{j}\right){\left(e_{ij}\cdot s_{i}\right)}^{3}\right), \end{array}$$

where the intensity of the dipole and quadrupole contributions are described using the functions *g*_1_,*q*_1_,*q*_2_:


[6] $$g_{1}\left(r_{ij}\right)=g\left(r_{ij}\right)+\frac{12}{35}q\left(r_{ij}\right),$$

[7] $$q_{1}\left(r_{ij}\right)=\frac{9}{5}q\left(r_{ij}\right),$$

[8] $$q_{2}\left(r_{ij}\right)=-\frac{2}{5}q\left(r_{ij}\right).$$

When modeling, it is convenient to describe the functions *q*(*r_ij_*) and *g*(*r_ij_*) with the Bethe–Slater curve:


[9] $$f\left(r_{ij}\right)=4\alpha {\left(\frac{r_{ij}}{\delta }\right)}^{2}\left(1-\gamma {\left(\frac{r_{ij}}{\delta }\right)}^{2}\right)e^{-{\left(\frac{r_{ij}}{\delta }\right)}^{2}}\Theta \left(R_{c}-r_{ij}\right),$$

where α (in eV), δ (in Å), and γ (dimensionless value) are constant coefficients that depend on the structure of the sample under study and Θ(*R_c_* − *r_ij_*) is the Heaviside function. The coefficients α, δ, and γ must be chosen so that the aforementioned function corresponds to the values of the magnetoelastic constant of the materials under consideration.

The following equation is used to calculate the spin temperature:


[10]

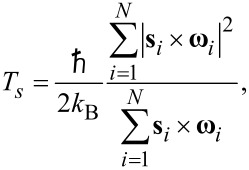



where **s***_i_* is vector representing the magnetic spin of the particle, ω*_i_* is the magnetic moment, and ℏ is the Planck constant. This approach to calculate spin temperature was proposed in [44].

The approach described in this paper and originally proposed by the authors [40] is implemented using direct simulation methods. At each time point, we do not know the assumed spin location, but we know its computed value, which is calculated based on empirical parameters or other previously obtained data. Therefore, an additional advantage is that systems of arbitrary size, including small ones, can be considered for calculating magnetic properties based on the combined model of molecular dynamics and magnetization dynamics.

The technique used includes simulations of atomic magnetic spins associated with lattice vibrations. The dynamics of these magnetic spins can be used to simulate a wide range of phenomena related to magnetoelasticity or to study the influence of defects on the magnetic properties of materials.

## Results and Discussion

As numerical experiments at the stage of modeling technological processes of niobium and cobalt sample manufacturing showed, the structure of the formed layers is not ideal. Visually, noticeable crystallization zones are observed in the formed nanofilms. In addition, there are areas of mixed structures, where the amorphous atomic structure most likely prevails. Quantitatively, the structure of nanofilms can be estimated, for example, by calculating the lattice ideality parameter [45]. This parameter is close to zero in ideal crystal lattices and has a positive value where the structure of the material differs from the reference one, and the higher the value of the parameter, the higher the degree of discrepancy.

**Figure 2:**
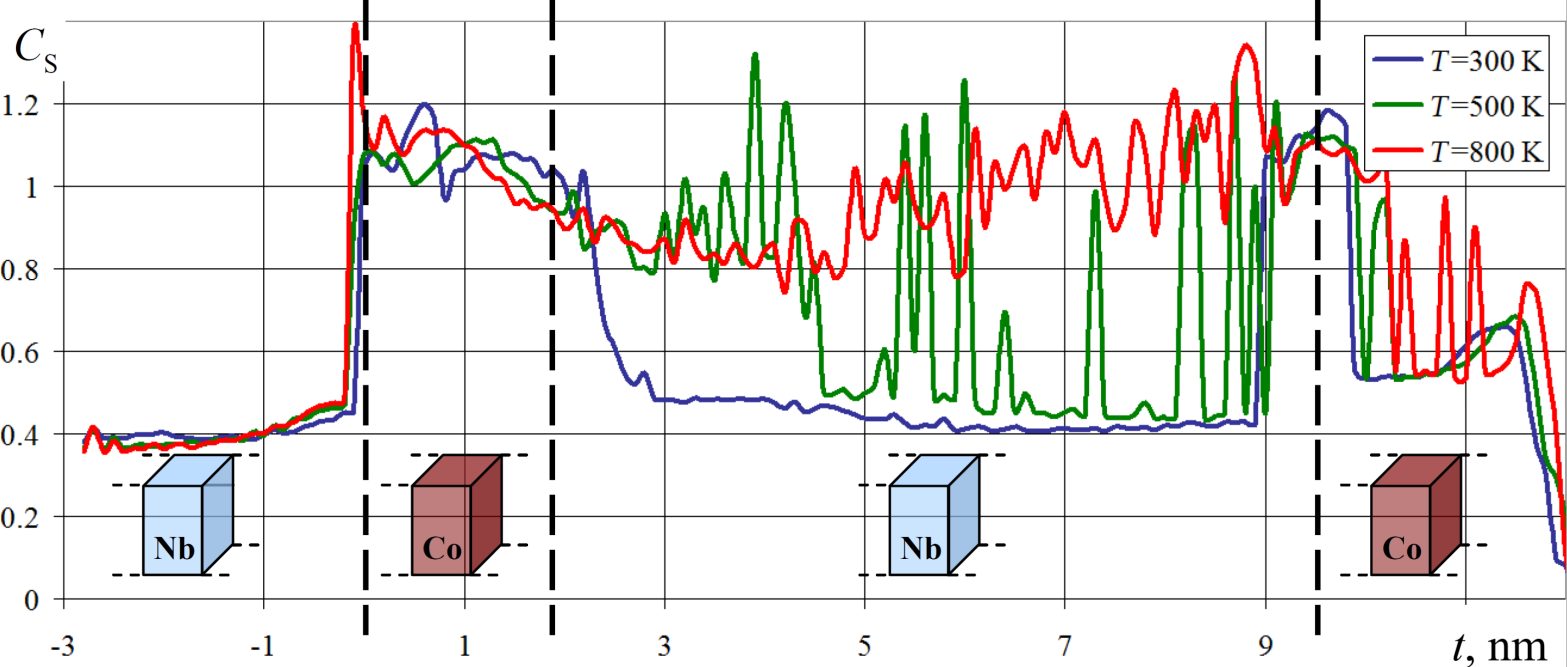
Variation of the average value of the crystal lattice ideality parameter in horizontal layers of a niobium and cobalt nanocomposite. Image reproduced from [37] (© 2020 A. Vakhrushev et al., distributed under Creative Commons Attribution License (http://creativecommons.org/licenses/by/4.0)).

For the sample under study, the change in the ideality parameter, averaged over thin horizontal layers, is shown in Figure 2. The legend to the figure provides information about the temperature of the substrate on which nanofilms were deposited in the numerical experiments.

**Figure 3:**
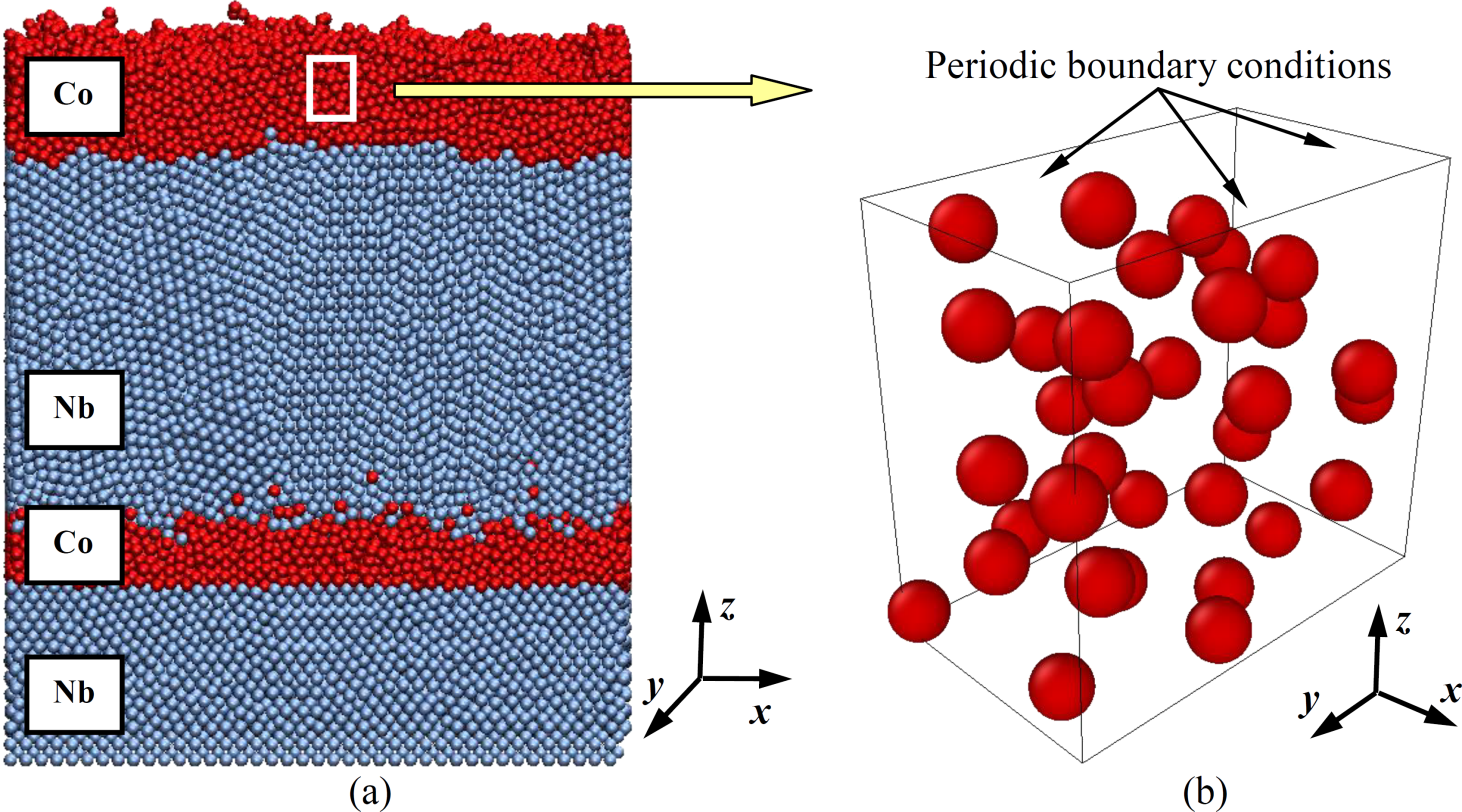
Multilayer nanocomposite of niobium and cobalt (a) formed in a numerical experiment during deposition on a 300 K substrate and a group of cobalt atoms was cut out to simulate the magnetic properties (b). The image shown in (a) was adapted from [37] (© 2020 A. Vakhrushev et al., distributed under the terms of the Creative Commons Attribution License (http://creativecommons.org/licenses/by/4.0)).

Niobium is known as one of the most actively used superconductors [46–47] with a superconducting transition temperature for pure metal equal to 9.25 K. In superconductors, including niobium, due to the Meissner effect, the phenomenon of complete or partial ejection of the magnetic field from the material volume occurs [48–49]. In the superconductivity mode, which is the mode of greatest interest for the magnetic behavior of the target film heterostructure, the absence of a magnetic field is observed inside the metal, which is predominantly concentrated near the surface. For the reasons previously described, niobium nanofilms were excluded from explicit consideration in numerical experiments to investigate the magnetic properties of the spin nanocomposite, whose appearance and structure are demonstrated in Figure 3а.

To investigate the magnetic properties of nanomaterials, the substrate temperature was set in the range of niobium nanofilm superconductivity mode operation at 5 K. Regarding the problem of nanofilm deposition and structure formation, we considered three substrate temperatures on which the deposition took place: 300 , 500, and 800 K. These temperatures are determined by process features of niobium and cobalt-based nanocomposite fabrication and can be seen in the legend shown in Figure 2. For both studies of magnetic properties and nanofilm deposition mechanisms, the substrate temperature was maintained using a Nose–Hoover thermostat.

Thus, at the initial stage of studying magnetic characteristics, the spin behavior of only cobalt atoms was analyzed for two calculation variants. In the first case, the cobalt atoms were located near the nodes of the hexagonal close-packed (HCP) crystal lattice, since this cobalt modification is more stable at temperatures up to 700 K. The functional features of the nanocomposite involve its superconducting niobium nanolayers, so the simulation was performed at a nanosystem temperature of 5 K. For the first version of the numerical experiment, a 2 × 2 × 2 unit crystal cell of HCP cobalt, bounded on all sides by periodic boundary conditions, was considered. The size of such a system (i.e., 0.5 nm × 0.87 nm × 0.82 nm) is relatively small.

For the second variant of the numerical experiment, the real structure of cobalt nanofilms obtained earlier by simulating their deposition processes was considered. In order to preserve the structure of the cobalt nanofilm, a small volume was cut out from it, shown in Figure 3a as a white rectangle. This volume had strictly the same dimensions as the ideal HCP structure in the first numerical experiment. A group of cobalt atoms with structural defects acquired as a result of film sputtering in an enlarged form is shown in Figure 3b. Henceforward, to simplify the formulation, the nanosystem of cobalt atoms from the numerical experiment with nanofilm deposition will be referred to as the real one.

The small size of the system in question was chosen for several reasons. First, the actual produced nanofilms in composites of cobalt and niobium have a small thickness, reaching 1–2 nm in some layers. Of practical interest are structural defects and their influence on the magnetic properties of thin films. Therefore, in our studies, a small volume in the cobalt nanofilm is cut out and the simulation results were compared to that of the corresponding volume with an ideal structure.

In addition, the periodic boundary conditions used in molecular dynamics enables one to balance the influence of direct boundary effects by symmetrically continuing identical computational volumes along those space directions where they are used, in our case along all three *x, y, z* directions. Lastly, the small computational cell in this work was used for clarity, so that the orientation of individual atom spins could be easily traced.

**Figure 4:**
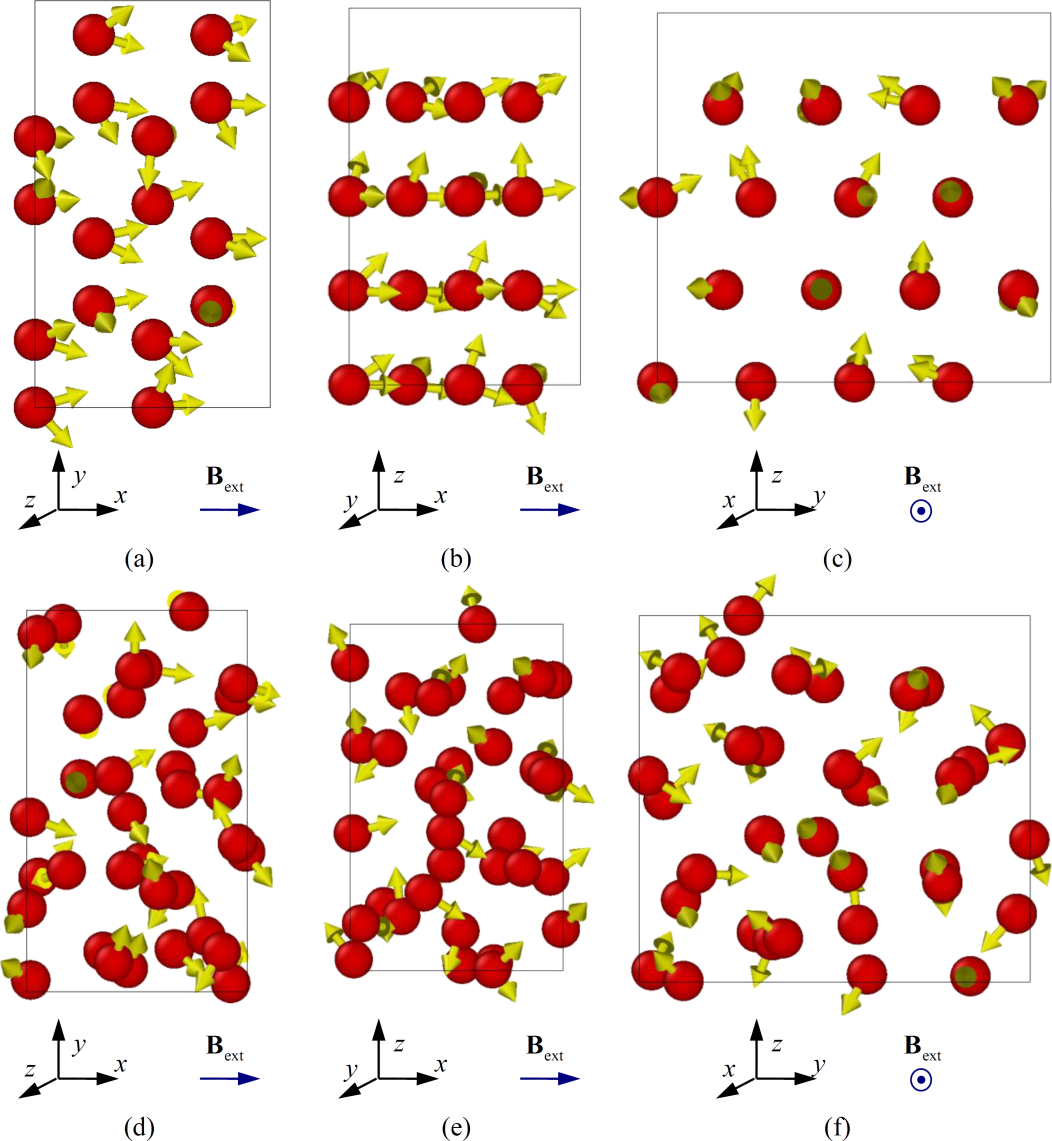
Spatial distribution of cobalt atom spins for ideal crystal hexagonal close-packed lattice (a), (b), (c) and nanofilm structure (d), (e), (f) formed in the numerical experiment at a deposition temperature of 300 K, spin relaxation time 100 ps, and external magnetic field value of 1.0 T.

Subsequently, the two selected systems were exposed to an external magnetic field with induction **B**_ext_ = 1.0 T in the *ox* axis direction (along the nanofilm surface for the real structure variant) for 100 ps. The result of the spin distribution at the final moment is shown in Figure 4. The time for the spin distributions of the atoms are shown in Figure 4 corresponds to 100 ps.

In order to catch the smallest changes in the spin behavior of the material and to take them into account, an integration step of 0.1 fs was chosen. The normal and spin temperatures were maintained at the initial value of 5 K. The coordinates of the atoms changed insignificantly, which is associated with small thermal fluctuations and their linear velocities. As for the spin rearrangement, at the initial times, corresponding to the interval of 0–5 ps, the change in the spin direction of atomic spins was active. At the initial time, a chaotic spin distribution regulated only by their initial spin temperature, was set for the atoms. Later, the direction of spins was influenced by the external magnetic field, as well as by their mutual arrangement and force behavior, which caused their reorientation.

Analysis of Figure 4 shows that there are significant differences in the spin distributions of an ideal crystalline hexagonal close-packed cobalt (letters (a), (b), (c)) and the nanofilm with structural defects formed as a result of the numerical experiment (letters (d), (e), (f)). Crystalline cobalt is characterized by small changes in spin states at finite times, with atomic spins set in the direction of external magnetic field induction, (i.e., *ox* axis). Nanofilms with structural defects and deviations from crystal lattice nodes are subject to higher randomness with respect to the direction of spins. The disordered orientation of spins is related to the enhanced influence of magnetic characteristics and forces of neighboring atoms. In the case of lattice distortions and defects in the material, zones of anomalies arise, which also bring about a stable magnetic state in the form of a local minimum of energy.

**Figure 5:**
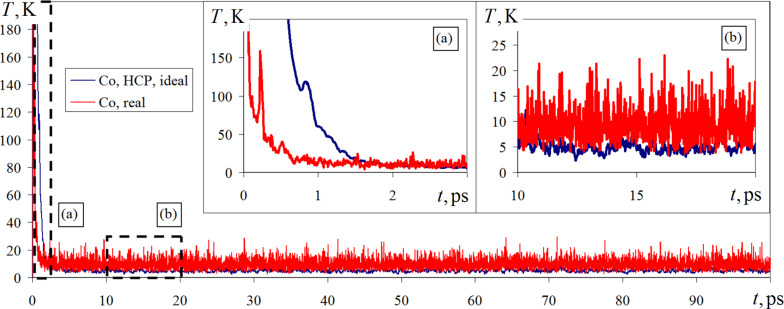
Changes in spin temperature under a constant external magnetic field of 1.0 T for ideal hexagonally close-packed cobalt and cobalt from the deposited nanofilm obtained in a numerical experiment.

Internally, the behavior of atomic spins can be evaluated by calculating the spin temperature of the material. The spin temperature is equal to the normal temperature but reflects the degrees of freedom of the atoms responsible for the magnetic energy fluxes. A graph of spin temperature variations for the two versions of ideal and real nanosystems under consideration is shown in Figure 5.

As it can be seen from Figure 5, at initial time intervals (0–3 ps) the spin temperature for both simulation variants is subject to considerable changes. In the graph of Figure 5, this time period is marked by the letter (a) and is shown in an enlarged form. The jumps in the spin temperature transformation at 0–3 ps correspond to an active rearrangement of the atomic spin directions, which were unstable in the initial state due to stochastic allocation. Subsequently, the spin temperature fluctuations decrease, and its fluctuations occur near the thermostat target value of 5 K. For an interval of 5–100 ps, the reorientation of spins is slow and mutually consistent, which is reflected in a small change in spin temperature. The system with a real structure has a less stable spin temperature behavior. The variation of this parameter in the range of 3–25 K indicates greater scattering and amplified oscillations of instantaneous values compared to those of the ideal structure case.

**Figure 6:**
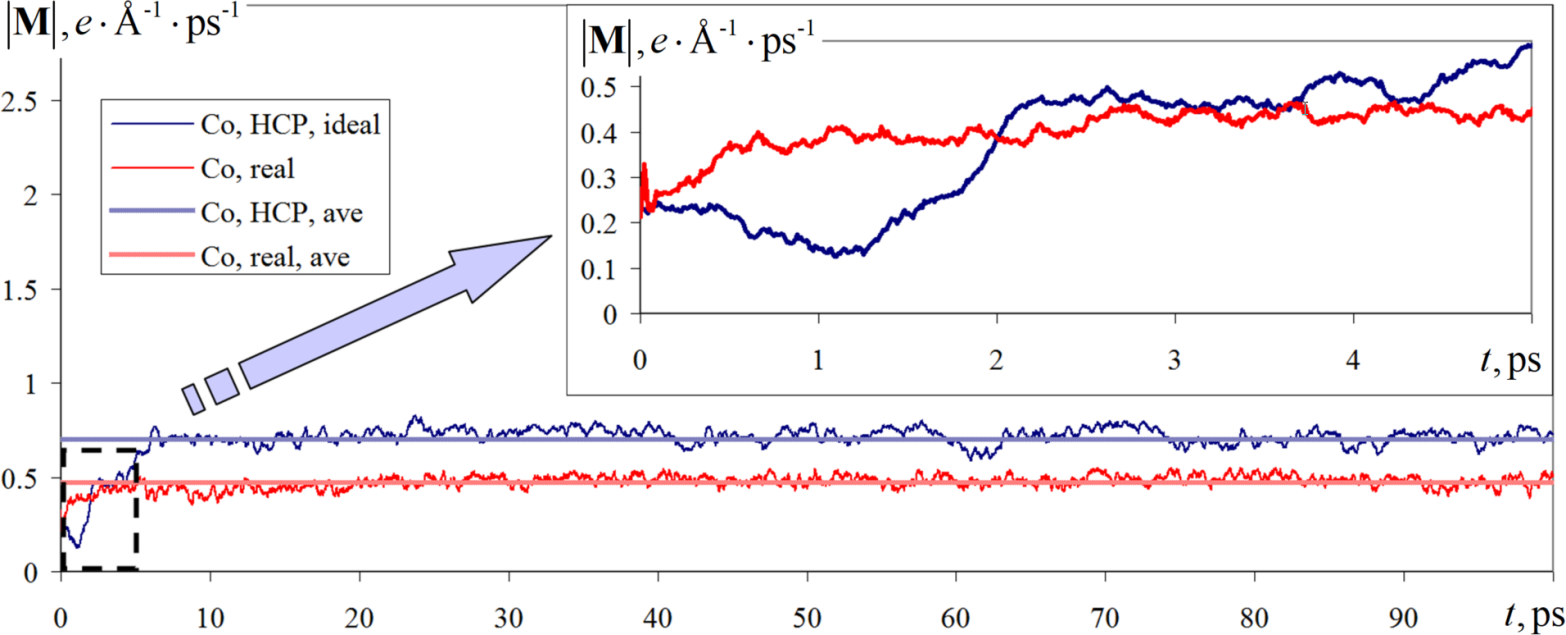
Changes in the magnetization vector modulus under a constant external magnetic field with an induction of 1.0 T for ideal hexagonally dense-packed cobalt and cobalt from the deposited nanofilm obtained in the numerical experiment.

Another macroscopic, but dependent on each atom, characteristic of the material is its magnetization. The magnetization determines the effect of partial or complete ordering of magnetic moments of a set of atoms under the influence of an external magnetic field, which allows the use of this value to evaluate the response of nanocomposites to external factors, considering its structure and internal features. Dynamics of the vector modulus of the investigated sample during simulation for two variants of the investigated structure under a constant external magnetic field of 1.0 T is presented in Figure 6.

The change in the modulus of the magnetization vector at the initial times (0–7 ps) is also characterized (e.g., spin temperature) by an increased variability. The gradual rearrangement of atomic spin states does not allow us to instantly find a stable energy state. The length of the initial section of the magnetization graph with high volatility has a longer length compared to the same value for the spin temperature.

For an interval of 7–100 ps, the magnetization modulus value is set near the mean value, which is 0.7 *e*·Å^−1^·ps^−1^ for the case of an ideal crystal structure and 0.47 *e*·Å^−1^·ps^−1^ for the real structure variant, where *e* is the notation of the electron charge. Such nanomaterial behavior is associated with the ordering of magnetic moments and is typical for ferromagnetic materials (e.g., cobalt [50–51]). Thus, from the analysis of the graphs in Figure 6 we can conclude that, despite the defects in the structure and the local arrangement of the atoms, cobalt retains its ferromagnetic character. However, there may be a decrease or deterioration of the magnetic macroscopic parameters, such as the magnetization modulus.

## Conclusion

A mathematical model capable of reproducing the time evolution of spin states and magnetic properties of a nanomaterial is proposed. This model reflects the response of an external magnetic field on the behavior of individual atoms, and considers the internal structure and features of structural defects at the nanoscale when calculating the macroscopic magnetic characteristics of a material.

The spatial distribution of cobalt atom spins for an ideal crystalline hexagonal close-packed lattice was studied. The structure of the nanofilm formed in a numerical experiment during deposition on a substrate maintained at a constant temperature of 300 K shows that the spin directions are significantly dependent on the material structure. Under an external magnetic field with an induction of 1.0 T, a reorientation of spins along the external magnetic field is observed for crystalline ordered cobalt. Conversely, for cobalt from the nanofilm a more chaotic distribution of spins is characteristic, but also with a predominant direction parallel to the vector of induction of the external magnetic field.

In numerical experiments, for the ideal and real structure it is obtained that after preliminary adjustment and significant jumps in the initial time intervals, the change of spin temperature occurs in a small range of values near the average thermostat target value. The system with the real structure has a less stable behavior of the spin temperature and a larger scattering of instantaneous values, which may indicate a less energetically stable state of the nanomaterial.

Analyses of simulation results show that for both calculation variants, with ideal hexagonal close-packed and with real structure, the ferromagnetic behavior is preserved for cobalt. Defects in the structure and local arrangement of atoms can be the cause of the deterioration of magnetic macroscopic parameters. For example, the magnetization modulus for the considered nanosystem in the case of the real structure decreased by 30–50%.

The mathematical model used in this work serves as a predictive tool, allowing to correct nanocomposite manufacturing processes and to reveal their weak points (e.g., the influence of indistinctly separated interfaces of nanofilms on the magnetic properties). Experimental studies on the subject of work are associated with a number of difficulties, and related results are planned to be published in following papers.

## References

[1] Markov S I, Shurina E P, Itkina N B (2019). J Phys: Conf Ser.

[2] Hardtdegen H, Mikulics M, Rieß S, Schuck M, Saltzmann T, Simon U, Longo M (2015). Prog Cryst Growth Charact Mater.

[3] Pasupathy A, Velraj R, Seeniraj R V (2008). Renewable Sustainable Energy Rev.

[4] Kazantseva N, Hinzke D, Nowak U, Chantrell R W, Atxitia U, Chubykalo-Fesenko O (2008). Phys Rev B.

[5] Wang Z, Wei J, Morse P, Dash J G, Vilches O E, Cobden D H (2010). Science.

[6] Lin Y-C, Dumcenco D O, Huang Y-S, Suenaga K (2014). Nat Nanotechnol.

[7] Chen H, Li J, Li Y, Cheng X (2020). J Alloys Compd.

[8] Wang Y, Tang B, Zhang S (2012). J Mater Chem.

[9] Barrio M, Font J, López D O, Muntasell J, Tamarit J L (1992). Sol Energy Mater Sol Cells.

[10] Bie Y, Li M, Malekian R, Chen F, Feng Z, Li Z (2018). Appl Therm Eng.

[11] Oikawa K, Ota T, Ohmori T, Tanaka Y, Morito H, Fujita A, Kainuma R, Fukamichi K, Ishida K (2002). Appl Phys Lett.

[12] Sutou Y, Imano Y, Koeda N, Omori T, Kainuma R, Ishida K, Oikawa K (2004). Appl Phys Lett.

[13] Hager M D, Bode S, Weber C, Schubert U S (2015). Prog Polym Sci.

[14] Müller I, Seelecke S (2001). Math Comput Modell.

[15] Ullakko K, Huang J K, Kantner C, O’Handley R C, Kokorin V V (1996). Appl Phys Lett.

[16] Owerre S A (2018). J Phys: Condens Matter.

[17] Paul S, Chatterjee N, Ghosal P (2019). J Syst Archit.

[18] Najam S, Ahmed J, Masood S, Ahmed C M (2019). IEEE Access.

[19] Zahnd G, Vila L, Pham V T, Cosset-Cheneau M, Lim W, Brenac A, Laczkowski P, Marty A, Attané J P (2018). Phys Rev B.

[20] Zhou T, Mohanta N, Han J E, Matos-Abiague A, Žutić I (2019). Phys Rev B.

[21] Serlin M, Tschirhart C L, Polshyn H, Zhang Y, Zhu J, Watanabe K, Taniguchi T, Balents L, Young A F (2020). Science.

[22] Das K S, Liu J, van Wees B J, Vera-Marun I J (2018). Nano Lett.

[23] Gurram M, Omar S, van Wees B J (2018). 2D Mater.

[24] Abert C (2019). Eur Phys J B.

[25] Shen R L, Zhong J (2009). Proc Inst Mech Eng, Part J.

[26] Yamamoto S, Taguchi M, Someya T, Kubota Y, Ito S, Wadati H, Fujisawa M, Capotondi F, Pedersoli E, Manfredda M (2015). Rev Sci Instrum.

[27] Meier F, Levy J, Loss D (2003). Phys Rev Lett.

[28] Lehmann J, Gaita-Ariño A, Coronado E, Loss D (2009). J Mater Chem.

[29] Kłobus W, Grudka A, Baumgartner A, Tomaszewski D, Schönenberger C, Martinek J (2014). Phys Rev B.

[30] Amigo N (2019). Mol Simul.

[31] Zong B, Phuoc N N, Wu Y, Ho P, Ma F, Han G, Yang Y, Li Z, He S, Wu Y (2015). ChemElectroChem.

[32] Kamilov I K, Murtazaev A K, Aliev K K (1999). Phys-Usp.

[33] Lewis L H, Marrows C H, Langridge S (2016). J Phys D: Appl Phys.

[34] Burr G W, Breitwisch M J, Franceschini M, Garetto D, Gopalakrishnan K, Jackson B, Kurdi B, Lam C, Lastras L A, Padilla A (2010). J Vac Sci Technol, B: Microelectron Nanometer Struct–Process, Meas, Phenom.

[35] Klenov N, Khaydukov Y, Bakurskiy S, Morari R, Soloviev I, Boian V, Keller T, Kupriyanov M, Sidorenko A, Keimer B (2019). Beilstein J Nanotechnol.

[36] Banerjee N, Ouassou J A, Zhu Y, Stelmashenko N A, Linder J, Blamire M G (2018). Phys Rev B.

[37] Vakhrushev A, Fedotov A, Boian V, Morari R, Sidorenko A (2020). Beilstein J Nanotechnol.

[38] Sidorenko A S, Morari R A, Boian V, Prepelitsa A A, Antropov E I, Savva Y B, Fedotov A Y, Sevryukhina O Y, Vakhrushev A V (2021). J Phys: Conf Ser.

[39] Vakhrushev A, Fedotov A, Sidorenko A (2021). Key Eng Mater.

[40] Tranchida J, Plimpton S J, Thibaudeau P, Thompson A P (2018). J Comput Phys.

[41] Beaujouan D, Thibaudeau P, Barreteau C (2012). Phys Rev B.

[42] Perera D, Eisenbach M, Nicholson D M, Stocks G M, Landau D P (2016). Phys Rev B.

[43] Neel L (1954). J Phys Radium.

[44] Nurdin W B, Schotte K-D (2000). Phys Rev E.

[45] Kelchner C L, Plimpton S J, Hamilton J C (1998). Phys Rev B.

[46] Finnemore D K, Stromberg T F, Swenson C A (1966). Phys Rev.

[47] Casalbuoni S, Knabbe E A, Kötzler J, Lilje L, von Sawilski L, Schmüser P, Steffen B (2005). Nucl Instrum Methods Phys Res, Sect A.

[48] Aull S, Kugeler O, Knobloch J (2012). Phys Rev Spec Top–Accel Beams.

[49] Thompson D J, Minhaj M S M, Wenger L E, Chen J T (1995). Phys Rev Lett.

[50] Moragues-Canovás M, Talbot-Eeckelaers C E, Catala L, Lloret F, Wernsdorfer W, Brechin E K, Mallah T (2006). Inorg Chem.

[51] Batallan F, Rosenman I, Sommers C B (1975). Phys Rev B.

